# Adipose tissue, but not skeletal muscle, sirtuin 1 expression is decreased in obesity and related to insulin sensitivity

**DOI:** 10.1007/s12020-018-1544-1

**Published:** 2018-02-07

**Authors:** Magdalena Stefanowicz, Agnieszka Nikołajuk, Natalia Matulewicz, Monika Karczewska-Kupczewska

**Affiliations:** 1 Department of Metabolic Diseases, Medical University of Bialystok, Bialystok, Poland; 2 Department of Prophylaxis of Metabolic Diseases, Institute of Animal Reproduction and Food Research, Polish Academy of Sciences, Olsztyn, Poland

**Keywords:** SIRT1, Obesity, Insulin resistance, Adipose tissue, Skeletal muscle

## Abstract

**Purpose:**

Sirtuin 1 may regulate glucose and lipid metabolism. We aimed to assess adipose tissue and skeletal muscle sirtuin 1 expression in relation to insulin sensitivity, the expression of proinflammatory and metabolic genes, and to study the regulation of sirtuin 1 expression by hyperinsulinemia and circulating free fatty acids elevation.

**Methods:**

We examined 60 normal-weight, 42 overweight and 15 obese young subjects. The hyperinsulinemic-euglycemic clamp technique was applied throughout to measure insulin sensitivity. In 20 subjects, two 6 h clamps were performed, one of them with concurrent Intralipid/heparin infusion. Biopsies of subcutaneous adipose tissue and skeletal muscle were collected for the measurement of gene and protein expression.

**Results:**

Obese subjects had lower adipose sirtuin 1 in comparison with normal-weight and overweight participants. Muscle sirtuin 1 did not differ between the groups. Adipose tissue sirtuin 1 was related to insulin sensitivity, adipose tissue *SLC2A4*. The relationship between adipose tissue sirtuin 1 and insulin sensitivity was still present after controlling for BMI, however, it disappeared after controlling for adipose tissue *SLC2A4*. Muscle sirtuin 1 was not related to insulin sensitivity. Hyperisulinemia decreased adipose tissue and increased muscle sirtuin 1 expression. Intralipid/heparin infusion negated these effects.

**Conclusions:**

Adipose tissue, but not muscle, sirtuin 1 is associated with insulin sensitivity in humans, possibly because of its correlation with adipose tissue *SLC2A4* expression. Insulin differentially regulates adipose tissue and skeletal muscle sirtuin 1 expression in the short-term and circulating free fatty acids elevation negates these effects, which may be associated with lipid-induced insulin resistance.

## Introduction

Sirtuin 1 (SIRT1), is a (NAD)-dependent deacetylase, a member of the sirtuin family [1]. SIRT1 can deacetylate many histone and nonhistone proteins. Therefore, it is involved in the regulation of multiple physiological processes, including substrate metabolism [1, 2].

SIRT1 may influence insulin signaling in multiple insulin sensitive cells [3]. It increases insulin receptor substrate 2 (IRS2) and protein kinase B (PKB, known as Akt) phosphorylation in response to insulin whereas it decreases the expression of protein-tyrosine phosphatase 1B (PTP1B), a negative regulator of insulin signalling [4–6].

Some studies indicate that SIRT1 enhances insulin signalling at least in part because of its antiinflammatory effects in AT. SIRT1 overexpression prevents macrophage accumulation caused by high-fat feeding [7]. SIRT1 represses proinflammatory gene expression in adipocytes, possibly through nuclear factor κB (NFκB) deacetylation and inhibition of binding to its target gene promoters [8]. It also increases adiponectin synthesis/secretion [9].

SIRT1 may also influence skeletal muscle metabolism through deacetylation of peroxisome proliferator-activated receptor gamma coactivator-1-α (PGC1-α), a mitochondrial fatty acid oxidation activator [10].

In the context of the above data, it was hypothesized that SIRT1 influences insulin sensitivity [11] and may be a therapeutic target in the prevention and treatment of disorders related to insulin resistance [9, 12, 13]. Resveratrol, a SIRT1 activator, had a protective effect against diet-induced insulin resistance in mice [5, 14] and decreased plasma glucose and triglycerides, homeostatic model assessment (HOMA) index and inflammation markers in humans with obesity [15]. However, in the studies with SIRT1 overexpression in multiple tissues in mice, both improved and unchanged glucose tolerance was observed [16, 17].

It remains unclear as to what the possible mechanism of beneficial SIRT1 action is on insulin sensitivity and which tissue is its major target. Furthermore, human data on the potential relationships of SIRT1 expression in different tissues with insulin sensitivity, especially with simultaneous assessment of SIRT1 in adipose tissue and muscle, are very limited.

Therefore, we aimed to assess AT and skeletal muscle *SIRT1* expression in young male subjects in relation to body weight, insulin sensitivity; tissue *SLC2A4* (encoding GLUT4), AT proinflammatory gene and *ADIPOQ* and muscle *PGC1A* expression. We also examined the regulation of tissue *SIRT1* expression by hyperinsulinemia and circulating free fatty acids (FFA) elevation.

## Materials and methods

### Study groups

We examined 117 healthy young men (aged between 18 and 35 years), 60 normal-weight, 42 overweight, and 15 with obesity. The exclusion criteria were: morbid obesity, cardiovascular disease, hypertension, peripheral vascular disease, infection or any other serious medical problem, smoking or the taking of drugs known to affect glucose or lipid metabolism. All participants had normal glucose tolerance according to World Health Organization criteria. All examinations were performed after an overnight fast. Body weight had been stable for at least the previous 3 months. Anthropometric and laboratory measurements were performed as described [18, 19].

The Ethics Committee of the Medical University of Białystok, Białystok, Poland, approved the study protocol. All participants gave written informed consent before entering the study.

### Insulin sensitivity

A 2 h hyperinsulinemic-euglycemic clamp was applied to assess insulin sensitivity [18]. Additionally, in 20 subjects, two 6 h clamps were performed, one of them with Intralipid/heparin infusion as previously described [19].

### Muscle and AT biopsies

Before the clamp, a vastus lateralis muscle and subcutaneous AT biopsy was performed. In a subgroup of 20 individuals, tissue biopsies were collected both before and after each clamp [19].

### Gene expression analysis

RNA isolation from tissues was performed as previously described [19]. *SIRT1* and *SLC2A4* mRNA expression in AT and skeletal muscle; *IL18*, *CCL2*, *IKBKB*, *NFKB1*, *NFKB2*, *RELA*, *MAPK8,* and *ADIPOQ* expression in AT and *PGC1A* mRNA expression in skeletal muscle was measured with Real Time PCR, using the Light Cycler® 480 II system (Roche Diagnostics GmbH) and Software release 1.5.0 SP3. Each sample was measured in triplicate. We used beta-2 microglobulin (*B2M*) or phosphoglycerate kinase 1 (*PGK1*) expression as a reference gene in muscle and AT, respectively. Primer sequences are shown in Supplementary Table 1.

### Western blot

Muscle lysates were prepared by tissue homogenization in a RIPA buffer (R0278, Sigma Aldrich, St. Louis, MO, USA) with added protease (PMSF, P7626, and Protease Inhibitor Cocktail P8340; Sigma-Aldrich) and phosphatase inhibitors (Pierce Phosphatase Inhibitor Mini Tablets 88,667; Thermo Fisher Scientific), and incubated for 1 h on ice. Thereafter samples were centrifuged and supernatants collected. Adipose tissue samples were prepared according to the manufacturer’s guidelines using the NucleoSpin®RNA/Protein Kit protocol (Macherey-Nagel GmbH, Duren, Germany). BCA Protein Assay Kit (Thermo Fisher Scientific) was used to determine protein concentration.

Samples were loaded into SDS-10% polyacrylamide gels. After electrophoresis proteins were transferred from the gel onto PVDF (polyvinylidene fluoride) membranes under wet conditions (30 V overnight at 4 °C). Membranes were blocked in Odyssey Blocking Buffer (TBS, LI-COR, Lincoln, NE) for 3 h at room temperature. Primary antibodies were carried out overnight at 4 °C with anti-SIRT1 (1:500, Abcam, Cambridge, UK) and anti-glyceraldehyde 3-phosphate dehydrogenase (GAPDH) (1:2000, Abcam). After thoroughly washing for 60 min with TBST 0.1% (Bio-Rad, Hercules, CA), the membranes were incubated with HRP-conjugated anti-mouse secondary antibodies (1:5000, Abcam) for 1 h at room temperature. After re-washing the membranes, immunodetection and visualization of proteins were performed on a ChemiDoc MP Imaging System (Bio-Rad) using chemiluminescent substrate detection reagent (ECL, Bio-Rad). GAPDH protein expression from parallel runs was used for the normalization of results.

### Statistical analysis

Statistical analysis was performed using the STATISTICA 12.5 Program (Statsoft, Krakow, Poland). Where variables did not have normal distribution, log-transformed values were used in the analyses. These variables were again anti-log-transformed to absolute values in the results for the purpose of data presentation. To analyze differences between the groups, one-way ANOVA and Tukey post-hoc tests were used. Differences before and after each clamp and between the 6 h clamps were assessed with Student’s *t*-test for paired samples. Correlations between variables were determined with the Pearson product-moment correlation and with multiple regression analysis. The level of significance was set at *p* < 0.05.

## Results

Subjects with overweight and obesity had higher fasting serum insulin and total cholesterol (all *p* < 0.05) than subjects in the normal-weight group. Additionally, subjects with obesity had higher LDL-cholesterol and triglycerides and lower HDL-cholesterol (all *p* < 0.05) than normal-weight subjects. Insulin sensitivity was similar in the normal-weight and overweight groups whereas subjects with obesity had lower insulin sensitivity in comparison to normal-weight and overweight participants (both *p* < 0.05) (Table 1).

**Table 1:** Anthropometric, biochemical, and metabolic characteristics of studied group (*n* = 117). Data are presented as mean ± S.D

	Normal-weight (*n* = 60)	Overweight (*n* = 42)	Obesity (*n* = 15)
Age (years)	22.8 ± 2.2	23.4 ± 2.5	24.5 ± 4.0
BMI (kg/m^2^)	22.6 ± 1.7	26.9 ± 1.5*	33.2 ± 2.9*^#^
Waist (cm)	82.2 ± 4.8	92.4 ± 7.0*	110.1 ± 9.2*^#^
Body fat (%)	14.5 ± 4.0	23.4 ± 5.4*	32.3 ± 7.5*^#^
Fasting glucose (mg/dl)	86.2 ± 8.1	87.9 ± 9.0	87.6 ± 8.7
Fasting insulin (μIU/ml)	9.5 ± 4.9	12.9 ± 6.8*	15.8 ± 5.1*
M_FMM_ value (mg/kg FFM/min)	7.1 ± 2.4	7.1 ± 3.0	4.4 ± 1.5*^#^
Total cholesterol (mg/dl)	161.8 ± 29.2	176.9 ± 33.9	186.3 ± 23.1*
Triglycerides (mg/dl)	81.2 ± 31.4	102.6 ± 55.2	118.7 ± 60.8*
HDL cholesterol (mg/dl)	59.8 ± 10.2	55.5 ± 8.4	51.6 ± 8.1*
LDL cholesterol (mg/dl)	95.9 ± 33.1	106.7 ± 30.8	120.0 ± 31.7*

*p < 0.05 for difference in overweight or obesity groups vs. normal-weight subjects; ^#^*p *< 0.05 for difference in the group with obesity vs. subjects with overweight

### AT and muscle gene expression

**Fig. 1:**
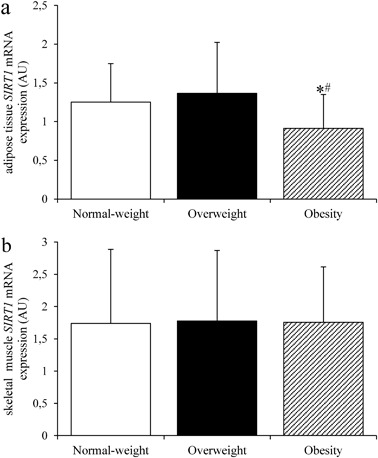
Adipose tissue (**a**) and skeletal muscle (**b**) *SIRT1* mRNA expression in the study groups (*n* = 117). Data are expressed as means ± S.D. *p < 0.05 vs. normal-weight subjects; ^#^*p < *0.05 in the obese vs. overweight subjects

AT *SIRT1* expression was lower in obese subjects than in normal-weight and overweight subjects (both *p* < 0.05) (Fig. 1a). Muscle *SIRT1* was not different among the study groups (Fig. 1b).

**Fig. 2:**
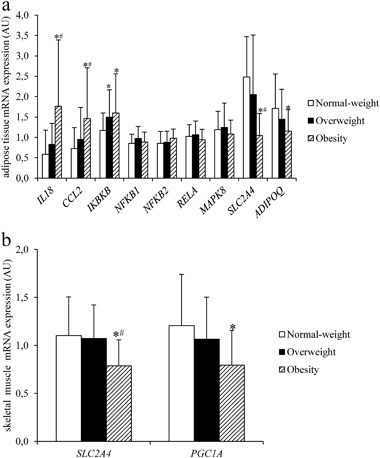
Adipose tissue proinflammatory gene, *SLC2A4*, and *ADIPOQ* mRNA expression (**a**) and muscle *SLC2A4* and *PGC1A* mRNA expression (**b**) in the study groups (*n* = 117). Data are expressed as means ± S.D. *p < 0.05 *vs*. normal-weight subjects; ^#^*p < *0.05 in the obese vs. overweight subjects

*IL18* and *CCL2* AT expression was higher whereas *SLC2A4* and *ADIPOQ* AT expression was lower in the obese than in normal-weight and overweight subjects (all *p* < 0.05, *ADIPOQ* only vs. normal-weight subjects). AT *IKBKB* was lower in the overweight and obese groups than in the normal-weight group (both *p* < 0.05) (Fig. 2a). Furthermore, in the obese group lower muscle *SLC2A4* in comparison to the normal weight and overweight groups and lower *PGC1A* (all *p* < 0.01) in comparison to the normal-weight group was observed (Fig. 2b).

### Correlations of tissue *SIRT1* expression with other parameters

**Fig. 3:**
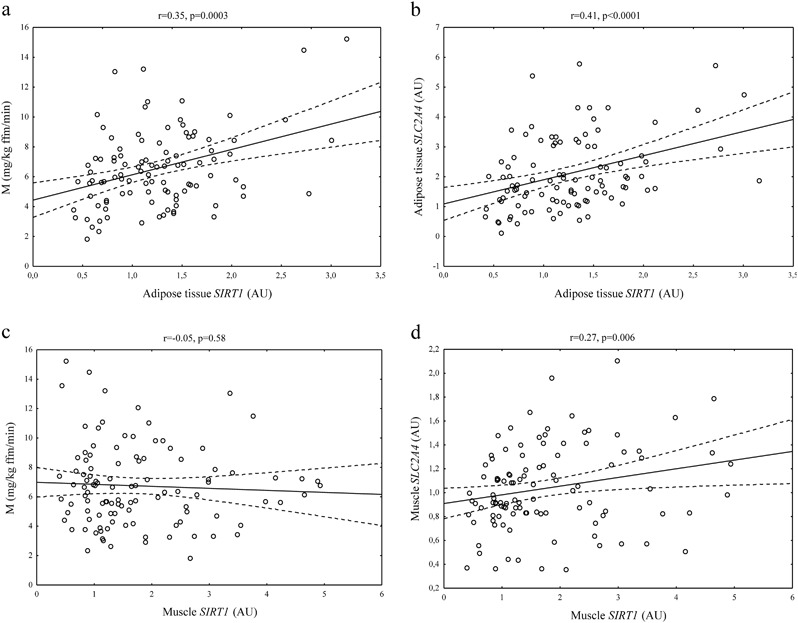
Correlations of adipose tissue and skeletal muscle *SIRT1* mRNA expression with insulin sensitivity (**a**, **c**) and respective tissue *SLC2A4* expression (**b**,** d**) in the entire study group (*n* = 117)

AT *SIRT1* expression was related to BMI, waist, percent of body fat, total cholesterol, triglycerides (*r* values between −0.23 and −0.29, all *p* < 0.05) and HDL cholesterol (*r* = 0.35, *p* < 0.001). We also demonstrated the correlation between AT *SIRT1* and M (*r* = 0.35, *p* = 0.0003; Fig. 3a). Furthermore, AT *SIRT1* was related to AT *SLC2A4* (*r* = 0.41, *p* < 0.0001; Fig. 3b), but not to inflammatory gene and *ADIPOQ* expression. Association between AT *SIRT1* and insulin sensitivity was still significant after controlling for BMI (*β* = 0.31, *p* = 0.006), whereas the correlation of AT *SIRT1* with BMI lost its significance after adjustment for M (*β* = −0,12, *p* = 0.22). However, the relationship between AT *SIRT1* and insulin sensitivity lost its significance after controlling for AT *SLC2A4* (*β* = 0.19, *p* = 0.053).

There was no correlations between muscle *SIRT1* and BMI, insulin sensitivity (Fig. 3c) and other clinical and biochemical parameters studied, however, we observed positive associations with muscle *SLC2A4* (*r* = 0.27; *p* = 0.006; Fig. 3d) and *PGC1A* (*r* = 0.20; *p* = 0.037). Muscle *SIRT1* was not related to AT *SIRT1* expression.

### Tissue protein expression

Due to the limited amount of tissue available, SIRT1 protein expression was measured in 10 AT (five from normal-weight and five from obese subjects) and eight muscle samples (four from normal-weight and four from obese subjects) in baseline conditions only. Both AT (*r* = 0.79, *p* = 0.007) and muscle (*r* = 0.80, *p* = 0.017) SIRT1 protein was strongly related to the respective mRNA expression.

**Fig. 4:**
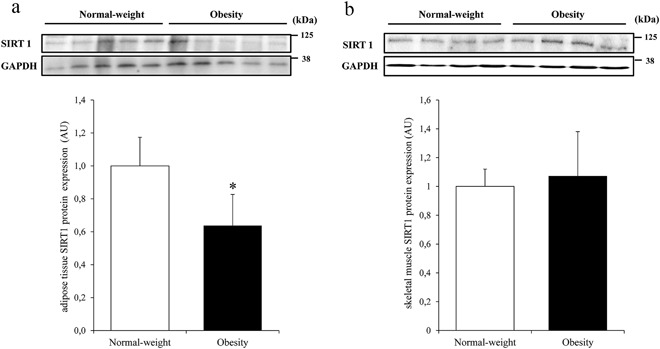
Western blot tests, with graph showing quantification by group. Adipose tissue (**a**) and skeletal muscle (**b**) SIRT1 protein expression in normal-weight and obese subjects. SIRT1 expression was normalized to GAPDH. Data are expressed as means ± S.D. *p < 0.05 vs. normal-weight subjects

AT SIRT1 protein was lower in obese subjects than in normal-weight subjects (*p* = 0.013, Fig. 4a) and was associated with M (*r* = 0.70, *p* = 0.023). Muscle SIRT1 protein was not different between the groups (*p* = 0.70, Fig. 4b). No correlation of muscle SIRT1 protein with M value was found (*r* = −0.37, *p* = 0.38).

### *SIRT1* expression in AT and muscle after 6 h clamp without and with Intralipid/heparin infusion

**Fig. 5:**
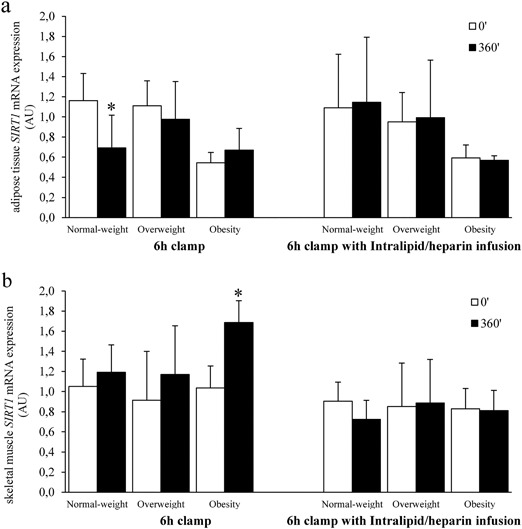
*SIRT1* mRNA expression during 6 h clamp without or with the concurrent Intralipid/heparin infusion in adipose tissue (**a**) and skeletal muscle (**b**) during the 6 h clamp (*n* = 20). Data are expressed as means ± S.D. *p < 0.05 vs. 0’ clamps

Hyperinsulinemia decreased *SIRT1* expression in AT by approx. 20% (*p* = 0.046). Subgroup analysis revealed that this effect was due to the change observed in the normal-weight group (*n* = 9, *p* = 0.046; Fig. 5a). The change in *SIRT1* after hyperinsulinemia (Δ*SIRT1*) differed between normal-weight and obese subjects (*p* = 0.021).

In contrast, muscle *SIRT1* expression increased after hyperinsulinemia (*p* = 0.016) in the entire study group. In subgroup analysis, this effect was observed mainly in the participants with obesity (*n* = 4, *p* = 0.013). However, muscle *SIRT1* expression increased in response to insulin also in seven out of nine normal-weight subjects, where it approached the level of significance (*p* = 0.09) and in five out of seven overweight subjects (Fig. 5b). In consequence, Δ*SIRT1* in response to insulin in skeletal muscle was not different between the study groups.

Intralipid/heparin infusion resulted in a more than fourfold rise of circulating FFA (*p* < 0.0001). Insulin sensitivity decreased by approx. 40% during the 6th hour of the clamp (*p* < 0.0001). No change in AT and skeletal muscle *SIRT1* expression was observed after Intralipid/heparin infusion (Fig. 5a, b).

## Discussion

In the present study we found that AT, but not skeletal muscle, *SIRT1* expression is decreased in obesity and is positively related to whole-body insulin sensitivity. We found that the relationship between AT *SIRT1* and *SLC2A4* explains the correlation of the former with whole-body insulin sensitivity. Furthermore, we observed that 6 h hyperinsulinemia decreased AT and increased skeletal muscle *SIRT1* expression and that both effects were negated by concurrent Intralipid/heparin infusion.

Our data indicate that AT *SIRT1* may be important for obesity-associated insulin resistance. Decreased AT *SIRT1* expression in obesity had also been reported in other human studies [20–25]. In some, obese subjects had BMI in the range of morbid obesity [22], whereas in others [20, 21] mean BMI was similar to the value observed in our obese group. Thus, it is interesting to note that overweight subjects in our study had an AT *SIRT1* expression comparable with those in the normal-weight group, which suggests that there could be a threshold associated with body fat accumulation, which determines a decrease in AT SIRT1. Our results suggest that such threshold may be associated with insulin resistance as overweight subjects had normal insulin sensitivity, which may be due to their young age. The relationship between AT *SIRT1* and indirect indices of insulin sensitivity [20, 21, 23] and the values from the clamp study in subjects with a family history of type 2 diabetes [26] was also observed by other researchers. In subjects with morbid obesity, decreased AT *SIRT1* was observed in the insulin resistant vs. insulin sensitive group [27]. We demonstrated that insulin sensitivity, and not BMI, was an independent predictor of AT *SIRT1*, whereas such analysis has not been reported in other studies.

Surprisingly, no differences in muscle *SIRT1* was found among the study groups. Individuals with type 2 diabetes had lower muscle SIRT1 protein compared to control subjects [4]. However, such difference may be secondary to diabetic conditions, as it was demonstrated in C2C12 myocytes that high glucose significantly reduces the number of SIRT1-positive nuclei and total cellular SIRT1 protein content [28].

We also did not observe any correlations between muscle *SIRT1* and metabolic parameters. Although muscle *SIRT1* was positively related to muscle *SLC2A4* and *PGC1A* expression, these associations did not seem to influence whole-body insulin sensitivity. Both increased and unchanged insulin-stimulated activation of Akt was observed in cultured myotubes with SIRT1 overexpression [4, 5]. Furthermore, studies with muscle-specific SIRT1 overexpression in rodents showed that it did not enhance insulin-stimulated muscle glucose uptake [29–31]. These data indicate that SIRT1 activation in tissues other than muscle may be important for modulating insulin action. Our data on the relationship between AT, but not skeletal muscle, *SIRT1* expression with insulin sensitivity, fall in line with this hypothesis.

Rutanen et al. [26] observed a correlation between AT *SIRT1* mRNA expression and insulin sensitivity in subjects with a family history of type 2 diabetes. They suggested that results obtained in AT reflected metabolic changes in skeletal muscle, as they observed positive correlation between AT and muscle SIRT1 expression in a small subgroup of their study subjects (*n* = 11) [26]. Although our results are generally in agreement with those of Rutanen et al. regarding positive correlation between AT *SIRT1* mRNA and insulin sensitivity, they do not support the hypothesis about the role of skeletal muscle *SIRT1* in determining insulin action. We also did not observe any correlation between AT and muscle *SIRT1* expression. This indicates that AT SIRT1 associates with whole-body insulin sensitivity without any relation to muscle SIRT1. In our study AT *SIRT1* was also not related to the local proinflammatory gene and *ADIPOQ* expression.

We found correlation between AT *SIRT1* and AT *SLC2A4* expression and this association explained the relationship between AT *SIRT1* and whole-body insulin sensitivity. SIRT1 knock down in adipocytes led to a decrease in GLUT4 translocation and glucose uptake after stimulation with insulin [8]. AT accounts only for a small fraction of whole-body glucose disposal, however, it was demonstrated that AT-specific GLUT4 depletion resulted in profound metabolic abnormalities, including muscle and liver insulin resistance [32]; whereas AT GLUT4 overexpression in mice with muscle GLUT4 knockout increased muscle glucose uptake [33]. The results of other studies that in part were similar to our own, also demonstrated a decreased AT*SLC2A4* expression in animal models of type 2 diabetes [34] and in insulin-resistant humans [33], which may serve to support our findings. Thus, SIRT1 may influence insulin sensitivity through its effect on adipose tissue GLUT4 expression. However, the cause-effect relationship cannot be established on the basis of our data and it is also possible that lower AT *SIRT1* expression in obesity may be an effect of hyperinsulinemia, as discussed below.

We next examined the effect of hyperinsulinemia and circulating FFA elevation on tissue *SIRT1* expression. In AT, hyperinsulinemia resulted in a decrease in *SIRT1*. AT SIRT1 increased in response to fasting [22] and weight loss [35], where a decrease in fasting insulin was also observed, however, no correlation between these changes was reported. On the other hand, an increase in AT *SIRT1* in response to weight loss without significant change in serum insulin was also observed [20]. It was demonstrated that SIRT1 expression in various tissues, including fat, increased after caloric restriction in rats, and that insulin attenuated this response [36]. To our knowledge, AT SIRT1 expression after hyperinsulinemia in humans has hitherto not been reported. Insulin stimulates adipogenesis and inhibits lipolysis, whereas SIRT1 exerts opposite effects, so the decrease in SIRT1 expression after insulin infusion might be important for the maintaining of adipocyte differentiation and function. It is interesting to note that this effect was present only in the normal-weight group. It is possible that mild prolonged hyperinsulinemia observed in obesity has already led to a decrease in AT *SIRT1* expression in the obese group and thus our experimental hyperinsulinemia did not promote any additional effect. We did not observe a decrease in AT *SIRT1* in response to insulin in the overweight group, despite baseline *SIRT1* and insulin sensitivity comparable to the normal-weight group. One may hypothesize that lack of response of AT *SIRT1* to insulin represents an early metabolic abnormality in this group, even before the onset of overt insulin resistance, measured as a decreased insulin-stimulated glucose uptake.

FFA elevation, obtained by Intralipid/heparin infusion, negated the insulin effect on AT *SIRT1*. This negation of insulin effect may seem paradoxical, when we take into account that a high-fat diet decreases AT SIRT1 [37], however, it may reflect insulin-desensitizing action of FFA and may indicate that AT SIRT1 response contributes to FFA-induced insulin resistance.

Muscle *SIRT1* expression increased after hyperinsulinemia. This effect was mostly expressed in the group with obesity, however, a similar tendency was also present in other groups. Insulin significantly increased SIRT1 expression in C2C12 myotubes under low glucose conditions, which was associated with an impaired insulin ability to exert myogenic-stimulating action [28]. However, in our relatively short-term, 6 h experiment, changes in *SIRT1* may influence substrate metabolism. Therefore, we can hypothesize that an increased muscle *SIRT1* expression during hyperinsulinemia might represent an additional mechanism to maintain muscle glucose uptake.

As in AT, Intralipid/heparin infusion negated the insulin effect on muscle *SIRT1* expression. Baseline tissue SIRT1 reflect rather long-term processes regulating its expression whereas the effects of insulin and FFA show short-term regulation of SIRT1 expression. Thus, although our data do not indicate muscle SIRT1 as a modulator of long-term insulin sensitivity, it is possible that it is regulated by short-term insulin fluctuations/nutrient availability to exert some metabolic effects.

The strengths of our study include: use of the “gold standard” in measurement of insulin sensitivity, i.e., the hyperinsulinemic-euglycemic clamp; analysis of AT and muscle gene expression in a human study with a large group of participants and the examination of an apparently healthy study population, which allowed us to assess early events in the development of obesity-related insulin resistance, without the confounding effects of hyperglycemia, morbid obesity and other diseases. Also, we for the first time report the relationship between AT *SIRT1* and *SLC2A4* as well as the tissue-specific effect of hyperinsulinemia on *SIRT1* expression.

One limitation of our study arises from the fact that limited tissue availability prevented SIRT1 protein expression being measured in samples from all participants. Nevertheless, we were able to demonstrate an excellent correlation between AT and muscle SIRT1 mRNA and protein expression. We were also able to validate the major findings of our study at the protein level in subgroups of participants, both in AT and in muscle. Another limitation of our study is the fact that one cannot establish cause-effect relationship on the basis of our results.

In conclusion, our data show that AT, but not skeletal muscle, *SIRT1* is associated with insulin sensitivity in healthy young humans, possibly because of its correlation with adipose tissue *SLC2A4* expression. Insulin differentially regulates AT and skeletal muscle *SIRT1* expression in the short-term and circulating FFA elevation negates these effects, which may be associated with lipid-induced insulin resistance.

## Electronic supplementary material

Supplementary Information
