# Supplementary material for: Adipose tissue, but not skeletal muscle, sirtuin 1 expression is decreased in obesity and related to insulin sensitivity
Source: Endocrine. 2018 Feb 7;60(2):263–71. doi: 10.1007/s12020-018-1544-1 (PMC5893655; doi:10.1007/s12020-018-1544-1)
Supplement: Supplementary file 1 — Supplementary Information [file 12020_2018_1544_MOESM1_ESM.docx]

Journal: **Endocrine**

Magdalena Stefanowicz^1^, Agnieszka Nikołajuk^2^, Natalia Matulewicz^1^, Monika Karczewska-Kupczewska^1,2^

**Adipose tissue, but not skeletal muscle, sirtuin 1 expression is decreased in obesity and related to insulin sensitivity**

^1^Department of Metabolic Diseases, Medical University of Bialystok, Poland

^2^Department of Prophylaxis of Metabolic Diseases, Institute of Animal Reproduction and Food Research, Polish Academy of Sciences, Olsztyn, Poland

**Corresponding author**

Monika Karczewska-Kupczewska, MD, PhD

Department of Metabolic Diseases, Medical University of Bialystok

Żurawia 71a, 15-540 Białystok, Poland

Phone: +48 85 7222558 Fax: +48 85 7222559

e-mail: monika3101@wp.pl

**Supplementary Table 1**

Assay Information

| Gene Symbol | Forward Primer Sequence | Reverse Primer Sequence | UPL Number |
| --- | --- | --- | --- |
| *SIRT1* | 5’-tgtacgacgaagacgacgac | 5’-ttcatcaccgaacagaaggtt | 63 |
| *IL8* | 5’-TAGCCAGGATCCACAAGTCC | 5’-CTGTGAGGTAAGATGGTGGCTA | 98 |
| *CCL2* | 5’-AGTCTCTGCCGCCCTTCT | 5’-GTGACTGGGGCATTGATTG | 83 |
| *IKBKB* | 5’-GGAACGCTGGACGACCTA | 5’-CTCGAGGTTTTTCCCTTAGTCTC | 24 |
| *NFKB1* | 5’-ACCCTGACCTTGCCTATTTG | 5’-AGCTCTTTTTCCCGATCTCC | 39 |
| *NFKB2* | 5’-CCCATCCATGACAGCAAAT | 5’-CTTGTCACAAAGCAGATAAACTTCA | 40 |
| *RELA* | 5’-ACGAACTGTTCCCCCTCAT | 5’-TGGGCTGCTCAATGATCTC | 21 |
| *MAPK8* | 5’-GGGAACACACAATAGAAGAGTGG | 5’-TGCCCCCGTATAACTCCAT | 25 |
| *SLC2A4* | 5’-GAGCAGGACAGGAGACAAGAA | 5’-AGAGTCTGCGTGGCAAGAAT | 95 |
| *ADIPOQ* | 5’-GGTGAGAAGGGTGAGAAAGGA | 5’-TTTCACCGATGTCTCCCTTAG | 85 |
| *PGC1A* | 5’-TGAGAGGGCCAAGCAAAG | 5’-ATAAATCACACGGCGCTCTT | 13 |
| *PGK1* | 5’-GGAGAACCTCCGCTTTCAT | 5’-GCTGGCTCGGCTTTAACC | 69 |
| *B2M* | 5’-CCGTGTGAACCATGTGACTTT | 5’-CCTCCATGATGCTGCTTACA | 117 |
